# The Hospital Nursing Supplement

**Published:** 1893-03-18

**Authors:** 


**"The Hospital" Nursing Mirror.**

Being the Extra Nursing Supplement of "The Hospital" Newspaper.

[Contributions for this Supplement should be addressed to the Editor, The Hospital, 428, Strand, London, W.C., and should have the word "Nursing" plainly written in left-hand top corner of the envelope.]

### News from the Nursing World.

### PRINCESS VICTORIA OF SCHLESWIG-HOLSTEIN.

We regret to announce the illness of H.R.H. Princess Victoria, daughter of Prince and Princess Christian, who is laid up at Buckingham Palace with severe cold and slight feverish symptoms. The Princess has been confined to her room since Friday last.

### THE NURSE DOLLS.

Our plea for the dolls has received most kindly and gratifying response from The Hospital readers. Many letters have come offering the assistance required. We beg that a little indulgence may be accorded to us in the matter of replying to our correspondents. When the distribution and other details are completed, we will announce in The Hospital that the small nurses are sorted out, ready to start on their travels in search of beautiful clean uniforms!

### A MODEST REQUEST.

Sister Katherine, of St. Mary's Home, Plaistow, is asking aid for her sick and destitute patients on such a modest scale that it is within everyone's power to comply with her request. She wants garments for the annual sale, the materials in each to cost 6½d. Who that has read of the work done by the nurses at Plaistow, and the terrible destitution they report, can refuse to contribute at least one useful garment to an association which requires and deserves all the help it can possibly secure?

### THE ROYAL FREE HOSPITAL.

The building operations at the Royal Free Hospital, Gray's Inn Road, will shortly be commenced, and many desirable improvements are contemplated. The accommodation for the nursing staff and the inadequate casualty department urgently need reformation. A great deal of excellent work is annually recorded at this hospital, which is admirably placed for freely helping the sick and the injured in this very poor neighbourhood.

### THE LIFE OF THE WARD.

What a pathetic picture was presented to us in the "Young Nurse's" letter last week. How lightly she describes the admission of herself and her sister at 16 and 17 years of age to institutional work. They were the "life of the ward," these two girls (we had nearly said these children), and at 19 the elder was considered, and no doubt thought herself, quite competent to be put in charge of 68 to 72 persons. "What the eye sees not, the heart grieves not," is a quaint old saying and a true one. Some women make better wives at 20 than others at 25, asserts our young friend. The trite remark is somewhat inconsequent, and the good wife and mother of 20 would probably prove better in both capacities if her parents persuaded her to postpone her marriage for a year or two. Although age may not give ability, it allows for the development of dormant talents and for the acquisition of what is even more valuable, namely, experience! We can only hope that we have left behind us the days when girls in their teens were considered "quite competent" to oversee large infirmary wards. Unfortunately it still seems the custom to admit very young nurses into some of the children's hospitals. We have a notice in our obituary this week of a promising young worker of 18. People think the nursing of children involves lesser trials of health and strength, forgetting, amongst other things, the preference of infectious diseases for young victims.

### CLAPHAM MATERNITY HOSPITAL.

Miss Helen Webb, M.B., was in the chair at the fourth annual meeting of the Clapham Maternity Hospital on March 9th. The general report and balance-sheet of the year were read by Dr. Annie McCall, who said that 206 patients had been treated in the hospital, 302 maternity out-patients attended, 26 nurses trained in monthly nursing, and 22 students in midwifery. The in-patients showed an increase of 54 over last year. At the St. John's Maternity branch department at Battersea, which was taken on last year, 68 patients were attended during the months of November and December. Funds are urgently needed for providing additional accommodation for patients at the Maternity Hospital, 41, Jeffreys Road, and an appeal for help was made at the meeting. At the close of the proceedings the hospital was inspected by numerous visitors, who appeared much pleased and interested with all they saw.

### FROM WASH-TUB TO WARD.

The transition is startling when it applies to persons, not things. Without doubt nurses should be good washers, but it hardly follows that laundresses are experienced in nursing. The nursing of sick paupers ought to be entrusted to as capable and experienced hands as the nursing of the well-to-do. The Totnes Board of Guardians have distinguished themselves by a selection which points, however, to a different opinion. An assistant nurse was required, and five applications were received. Of the fitness of the other candidates to take charge of sick paupers nothing is said, but Elizabeth Davis, at present laundress in the Workhouse, was elected. Is Elizabeth Davis a nurse whom "reduced circumstances" have driven to the wash-tub; and is she returning to her original calling? Surely nothing else can explain the appointment.

### LIBERALITY AT LANCASTER.

The Committee of the Lancaster Infirmary announced at their annual meeting, that tenders for the new building have been accepted and the work commenced. Therefore we may hope within a reasonable period to find this large and populous district provided with more adequate hospital accommodation. Of the need for this no visitor to the old infirmary can entertain any doubt. The working men of Lancashire set a good example to other places, for they support their infirmary nobly; their subscriptions last year amounted to close on £500. Amongst other donations, that of £70 from the North-Western Football League was reported.

### DIFFICULT NURSING.

It being generally conceded that "to serve two masters" is a difficult task, our readers will surely bestow a little pity on the nurses at Bath who are thus circumstanced. The medical officer lately appealed to the General Purposes Committee of the Bath Board of Guardians on the following point. He asked, says the *Bath Herald*, "whether the master" (of the workhouse) "had any right to place extra duties on the nurses under him without first asking his opinion upon the point, and if so, whether the master had any right to put it in force without the consent of the Board being first obtained?" It sounds a most pertinent query; but instead of any definite decision as to which gentleman is actual head of the nursing department, it is settled that in future all alterations of nurses' duties shall "be first reported to the guardians, and their sanction obtained before being carried into effect." Is there no matron or superintendent of nurses at Bath? If so, her office must be a sinecure. Any change in a nurse's work is generally caused by some emergency; it will therefore be interesting to know whether the special demand of acute cases of illness, operations, &c., have to be postponed until a board meeting takes place.

### SICK AND HUNGRY.

There is great distress in Lancashire just now, and the long-continued strike at Oldham has caused many mills to stop working. What this means can perhaps be only fully estimated by those who live amongst and nurse the poor in these districts. One nurse writes of "the hungry little faces around" her, and tells of hard-working mothers driven by famine to part with sewing machines and other of their possessions. Hunger brings sickness in its train, and there have been many cases of influenza in Lancashire, although happily thus far of a mild type.

### ENLARGEMENT AND IMPROVEMENT.

Important additions to the Taunton and Somerset Hospital are contemplated, which will include a children's ward, and also a new wing to provide proper accommodation for the nursing staff. Two cottages are to be converted into an isolation block, which is certainly a most desirable and needful addition to an institution. The work is to be carried out as a memorial to the late Mr. Wm. Liddon, F.R.C.S., for many years honorary surgeon to the hospital in which he always took the keenest interest.

### BANISHED BEDS.

The modern probationer does not realise many of the trials which formerly beset the path of her sisters in the profession. Amongst these may be classed the flock beds, which were once so general. The patients, poor things, hitherto accustomed to thin, lumpy, and frequently unclean bedding in their own homes, accepted thick flock as a welcome improvement. Intelligent nurses disliked it, knowing its drawbacks for sick people, from their professional point of view; and certainly indolent nurses cordially hated beds which were troublesome to make, and seldom looked neat five minutes afterwards. How glad the nurses at Hull must be to have hair mattresses at last, and, as we read last week, to have banished their old, but not beloved acquaintance, the flock bed.

### ST. PATRICK'S HOME.

This association, for supplying trained nurses to the sick poor of Dublin in their own homes, which is affiliated with the Q.V.J.I., is doing most excellent work. The services of the nurses are given to needy patients of all denominations, and a night attendant is provided when necessary. In asking for increased financial support the committee say, "The work of the institution is a benefit to the whole community, skilled nursing tending to shorten the time during which the patients are absent from their employment, and raising the standard of the people by teaching them the advantages of order, cleanliness, and fresh air."

### SHORT ITEMS.

The new home for the district nurses at Aberdeen is complete, and many friends assisted in the furnishing by gifts of useful articles. The District Nursing Dorcas Society has contributed funds and materials to the association, and a branch of the Scottish Needlework Guild is now being established in Aberdeenshire.—It is proposed to start a Nursing Institute at Shepton Mallet, and to engage a nurse for six months as a commencement.—The sixth annual meeting of the Royal National Pension Fund for Nurses was held recently, and the report showed that 89 nurses had received sick pay during last year. Hospitals and other institutions employing nurses evince increasing interest in this useful fund.—One of the omnibus proprietors at Worthing kindly allows the district nurses free conveyance, the area over which their duties extend being a very wide one.—The Bromyard District Nurse's offer to give a course of "Sanitation and Nursing" lectures to the women in that neighbourhood has been gladly accepted.—The agency for fully-trained nurses on co-operative principles has been established at 46, Sardinia Terrace, Glasgow.—The new Home for Nurses at Chipping was recently formally opened. Amongst the speeches General Hale's was specially appropriate, for in praising the work already done by the nurses in the neighbourhood, he advised the younger nurses to continue loyal to their principal and devoted and punctual in work, thus securing comfort for all.

### THE DISABLED NURSE.

Since last week we have received 5s. from F. N. L., 10s. from Policy 79, and 1s. from Policy 33, making a total of £11 15s., of which £2 17s. 6d. has been paid to the invalid nurse.

### "THE HOSPITAL" ENDOWED BED.

Our readers will remember a letter which appeared in our columns on January 14th of the present year, containing a proposal from F.L.E. that the nurse's bed should be permanently endowed. A thousand nurses were invited to collect 30s. each. In the following week (January 21st), we expressed our willingness to hear from nurses in favour of the plan, asking for communications to reach us "on or before March 14th next." In reply, we have received sixteen offers to collect £1, two or three correspondents adding the words "and upwards," one offer to collect 10s., and from F.L.E. a letter, saying "You may put my name down for £12, and I hope it will be more." The whole sum actually promised is therefore, roughly speaking, £28 10s. towards the £1,500 suggested by our first correspondent. But there is another side to the matter, for the sum needed to carry on the original plan is, as our readers know, £30 per annum. Towards this we have received only £13 5s. for the current year, £16 19s. is therefore still required if the bed is to be supported during 1893. The permanent endowment of the bed has evidently not commended itself to our nurse-readers, and probably many have withheld their subscriptions pending the possible change in the arrangements. As the endowment scheme is not immediately practicable, we trust that those nurses who kindly offered to collect, will send us the amount of their collections to carry on "The Hospital" bed under the annual arrangement. Last week the subscriptions should have appeared, as: Miss Yorke, £1, and G. S., 5s. We have since received 5s. from a Queen's Nurse.

### Management of Consumption.

### VI.—THE TEMPERATURE OF CONSUMPTION.

The importance of taking an accurate temperature in consumption can hardly be over-estimated. The temperature forms one of the surest guides the medical man possesses, which tells him almost infallibly whether the case is doing well or badly. The nurse has it in her power to make this test of progress either altogether accurate or altogether misleading. A temperature registered without due precautions is valueless, whereas one taken carefully is of the utmost importance.

Many different types of temperature may be met with in consumption, but they all, for the most part, conform to the hectic type; that is, the temperature in the evening goes up to a certain height, say 100 deg. to 101 deg., and then in the morning it is found to be normal again, and this goes on indefinitely; but however high it rises in the evening it always drops somewhere near the normal line in the morning. The more acute and rapid the disease in the lungs, the higher are the evening freaks of temperature on the chart; and the longer are the lines joining the morning and evening temperature. Sometimes the evening temperature may rise to 104 or even 104·8 deg., but it has scarcely ever been observed higher than 105 deg. This hectic form of temperature may be conveniently divided into three classes—(1) The subfebrile class, when the temperature does not reach higher than 100 deg.; (2) the moderately febrile, when it is as high as 102 deg.; and (3) the febrile, when it reaches higher than 102 deg. Broadly speaking, when the temperature in consumption is hectic, it means that the tubercular process in the lungs is on the march, and the height of the temperature is a measure of the intensity of the process. Sometimes the temperature is almost normal, although the patient is going from bad to worse. When from some course or other the process is very acute, or when there is a general deposition of tubercles all over the lungs or other organs, the temperature takes on what is known as a continued type, that means, that it keeps up, and although there are morning remissions, yet it never comes anywhere near normal; perhaps it may remain somewhere between 101 deg. and 103 deg. for two weeks or more. In very bad and acute cases a peculiar feature of the temperature is often observed. Instead of the evening temperature being highest, things are reversed—the morning being highest, and the evening temperature being lowest. This is a sign of bad omen, and generally a fatal termination is not far distant. This reversal of the order of things is called *Typus Inversus*. So long as the temperature keeps up, however bad the case may be, death will not take place; but when the temperature shows signs of falling, and when after having been high for some weeks it comes down to the normal either suddenly or gradually, then a fatal termination may be expected.

It has been before said that after an attack of hæmoptysis the temperature rises and keeps up for a day or two at a somewhat high level, and then gradually falls again. When pneumothorax occurs, the temperature falls below normal, and remains there for a short time, and then when pleurisy has set in, it rises higher than before.

It may not be out of place to say a few words concerning the taking of temperatures. The temperature may be taken in the mouth, the axilla, or the rectum. In consumption the mouth is a dangerous place, on account of the liability of the patient to cough during the process. To take a temperature in the axilla properly, requires more care and trouble than anywhere else, and the temperature cannot always be taken in the rectum. If care and patience be exercised the mouth is the best place to take the temperature in, and if the patient be properly cautioned there is no reason why he should break more thermometers than an ordinary patient. The bulb of the thermometer should be buried under the tongue, and left there with the mouth closed for five minutes. But the nurse must be on her guard in this, or fallacies of a startling nature are apt to creep in. If the patient has been walking in the cold air with his mouth open, or has been coughing or talking, or if he has taken a bath, or has just been brushing his teeth or drinking cold fluids; then the thermometer will register a very low temperature; in some cases it cannot be made to go higher than 97 deg. This often accounts for the lowness of morning temperatures. So it is a good rule never to take the temperature until an hour or so after washing or eating in the morning, and immediately before doing it to see that the patient keeps his mouth closed for five minutes. In registering the temperature on the chart, care should be taken to make neat dots and draw thin, straight lines, as there is nothing which adds so much to the neatness of the ward, and brings more credit to the nurse, than a well-kept temperature chart.

When the temperature is at all high it is well to take it every four hours, and by so doing all but the most trifling variations are recorded. Sometimes the temperature rises so high that treatment of some kind has to be adopted to combat it. Of the various drugs which depress the temperature the most common are quinine, antipyrin, salycilate of soda, and the far famed Niemeyris pill, which consists of digitalis, opium, and quinine; this, if given frequently, say one pill, three, or four times a day, rarely fails to reduce the temperature.

When giving these drugs it becomes a matter of importance to always administer them an hour or two before the temperature is at its highest; in this way a high temperature is avoided, and what benefit is likely to accrue to the patient from a reduction in temperature will come. It must not be supposed, however, that by simply reducing the temperature the case is thereby much improved; from the point of view of comfort such is undoubtedly the case, but it is of frequent occurrence to observe patients with the temperature reduced to normal, and yet steadily losing flesh.

Sometimes it is necessary to reduce the temperature by means of wet packing; this is, however, something like using a steam hammer to crack a filbert.

The important thing for the nurse to know, is how to take the temperature correctly, beyond this there is perhaps not much scope for the display of her powers.

### Minor Appointments.

St. Mary's Cottage Hospital, Tenbury.—Miss Mary A. Nott, who was trained at the General Hospital, Birmingham, has been appointed Nurse in Charge of St. Mary's Cottage Hospital, Tenbury. Miss Nott's testimonials very good, and we wish her success in her new work.

### Derby County Asylum.

### ST. JOHN AMBULANCE EXAMINATION.—FIRST AID CERTIFICATE.

The following nurses and attendants, 41 in number, successfully passed the St. John Ambulance Examination on February 21st, the result of which was made known on March 6th:—

Nurses.—Wade, Longden, Smith, Ghilks, Hyett, Pearson, Evans, Hall, Savidge, Hopkins, Parry, Butler, Fletcher, Edwards, Kinsella, Fancourt, Kirke, Ward, Head Nurse Mary Withers, Housekeeper Annie Newport.

Attendants.—Dean, Curtis, Presland, Markham Heaton, Cooper, Davis, Twigg, Metcalfe, Plummer, Thorold, Blood, Caborne, Parriss, F. Harrison, Speck, Hutchinson, Borrill, Stanley, Hardy, Travis, Head Attendant Harry Bird.

Fifty-eight members of the staff went in for examination, of whom 55 successfully passed, including the two head attendants, and all the nurses and attendants who were candidates. Three were rejected, on account of having failed to satisfy the examiner, Dr. J. W. Martin, Sheffield.

most advisable to the benefactor, and constitutes a very useful provision for the future without danger of waste. It is perhaps not out of place here to call attention to a very touching instance of disinterestedness on the part of one of our nurses, Sister Emma Durham, who received £200 for services rendered as hospital-trained nurse to the late Lord Tennyson, and has contributed the whole of the money to the "Junius S. Morgan Benevolent Fund," for the sole use and benefit of the poorer nurses, in commemoration of the illustrious man whom she had attended in his illness.

The Quinquennial Valuation.

An allusion will be found in the report to the quinquennial valuation. This being the first of the kind since the commencement of our Society has required very great care; and as the whole of the work has to be organised from the beginning, our actuary, Mr. George King, is very actively engaged upon the matter. Upon his report necessarily must depend the apportionment of the bonuses, and the principle which the committee can adopt in making them. It is necessarily a work of time, and I cannot promise a very early solution of it; but it is engaging the fullest attention both of the officials and the Council, and no time will be lost in communicating to the members of the fund the decisions as soon as they can be arrived at. In the meanwhile it may be of interest if I state to the members of the fund that, notwithstanding the depression and difficult financial period in many respects through which the money markets have passed in the last five years, we have not to deplore a delay or default in a single instance in the interest upon our investments, and at a valuation made by myself very recently I found that the capital value of the fund was quite intact.

Steady Progress.

Our business is steadily progressing, and when I reflect that in less than five years we have over 2,400 nurses insured, and that our appeals are necessarily made to that single class of the community which is not a very rich one, I think every one must admit that the progress made is simply astonishing and out of proportion with the results obtained by any other insurance company in the first five years of its existence. The reason for this is twofold. In the first place we give the insured not only the whole of the profit made, without any charge for management, except actual clerical disbursements, rendering the institution mutual in its benefits; but, secondly, we add a considerable bonus, which has been provided by liberal contributions of well-wishers to the nurses, and swells their fund without cost to themselves. I am also aware that, in appealing to the nurses to insure their lives and benefit by what we offer them, we have the advantage of addressing a particularly intelligent and well-conducted class of the community, who can understand, quite as well as the Council do, all the advantages we offer. And to the above reasons I feel certain we owe our past success. My confidence in the steady growth of the business is assured. I think the last five years have proved that not only is the fund doing good, but that it was very much needed, and that the benevolent gentlemen who contributed to its establishment—and our founder, Mr. Burdett, who organised the idea—are all entitled, not only to the gratitude of the nurses, but also to that of the community at large. In concluding my remarks, I desire to place on record the appreciation of the Council for the excellent services rendered by their secretary, Mr. Dick, their assistant secretary, Mr. Pocock, and the other members of the staff, who have all contributed, in the measure of their power, to the satisfactory results of the past year.

Election of Representatives of Annuitants and Policyholders.

Mr. George King reported that Dr. Potter and himself (the scrutineers) had gone through the votes, and found that the ladies recommended by the Council had been elected—viz.: Miss C. Davidson, Royal Infirmary, Liverpool; Miss E. Fisher, the General Infirmary at Leeds; Miss L. M. Gordon, St. Thomas's Hospital; Miss K. H. Monk, King's College Hospital; Mrs. G. Q. Roberts (late) London Hospital; Miss A. Ross, Victoria Infirmary, Glasgow; Miss E. Vincent, St. Marylebone Infirmary. The smallest number of votes for any name on this list was 1,011; the largest, 1,048. A number of other ladies had a few votes cast for them, but the largest number was 12. So the majority is overwhelming. It is surprising how many vote. It shows the interest taken in the matter.

The following retiring members of the Council were re-elected: Dr. J. S. Bristowe, Messrs. Henry C. Burdett, E. Murray Ind, Percival A. Nairne, and Clifford Wigram. On the motion of the Chairman, the auditor, Mr. Frederick Whinney (of the firm of Messrs. Whinney, Hurlbatt, and Smith, chartered accountants) was re-elected, the remuneration to be at the same rate as before—twenty guineas.

Thanks to the Chairman.

Mr. Burdett: I propose a vote of thanks to the chairman. This is not at all a formal vote, as I said on a previous occasion. The chairman gives a great amount of time to the affairs of the Fund, and renders great services. He has given the best testimonial of the success of the work in announcing that, despite all the depression, the whole of our securities stand without depreciation of any kind, after the close investigation he has recently made. The chairman takes a great personal interest in the investments, and looks after them closely; and, though he helps us in many ways, that is perhaps the most important work he does for us. His time, influence, and knowledge have been at the service of the Fund, and have proved a most substantial help. His post is not a sinecure. It involves regular attendance, many details of work, and a great deal of troublesome business. We have to thank him not only for his conduct in the chair to-day, but also for his counsel and advice on all occasions. Mr. Cotes seconded the vote, which was carried unanimously.

The Chairman, in reply, said: I am extremely obliged to you, as well as to Mr. Burdett and Mr. Cotes, for your kindness in thanking me for what, after all, is a labour of love on my part, and gives me fully as much pleasure in carrying out as it can afford yourselves if you find it successful. I don't think we have any reason to regret the course entered upon from the first day that we started this fund on the suggestion of Mr. Burdett, aided by my father-in-law, Mr. Morgan, Lord Rothschild, Mr. E. A. Hambro, and Mr. Henry Hucks-Gibbs, who were the fathers of the Fund. There is no day we do not feel that the future is more promising even than the past. I can scarcely say the extent to which this Fund may go in the future, and the good that it may do. It is the most beneficent fund for nurses, looked at financially, which has ever been started, and it is, perhaps, one of the most important institutions in the City of London. There are over £140,000 invested already, after scarcely five years of existence, two of which were occupied in the elementary stages, getting Bills and Acts to enable us to go to work. As long as I have strength and ability I shall do all I can to make this institution a success.

### Appointments.

Bristol General Hospital.—Miss Sophie Morris has been appointed Matron of this hospital. Miss Morris was trained at the General Hospital, Birmingham, and has previously held the posts of Night Superintendent at the Bristol Royal Infirmary and the Bristol General Hospital, having subsequently been appointed Assistant Matron at Bristol General Hospital. Knowing as we do the care exercised in the management of this institution, we congratulate Miss Morris upon the high compliment she has thus received, as well as the committee, who have shown a wise discretion in giving this important appointment to a lady whose work is so well known and appreciated in the city of Bristol.

### Night Nursing.

Why should "night duty" be so often spoken of with either dislike or contempt? Probably in the first case the nurse does not feel well, and does not take her food with good appetite, and thus she naturally objects to those months during which she has to turn day into night and night into day. As regards health, it is certainly those who are light sleepers who suffer most. Even when the dormitories are, as ought certainly to be the invariable rule, quite apart from the rest of the building, there are always unavoidable noises which penetrate from the world outside the walls. By day the city is awake; whether it be a big town or a small one, an incessant sound or variety of sounds fall on our ears. When the hours of daylight are the only ones in which a night nurse can legitimately sleep, it behoves herself and those responsible for her well-being to afford every possible facility for quiet, absolute stillness in her vicinity. Hence separate bed-rooms are perhaps more essential, if possible, for night nurses than for those on day duty. Everything should be done to encourage the habit of securing regular sleep, and often the nurse who goes straight to bed after the meal which follows twelve hours' hard work falls into a state of oblivion long before her neighbour has made up her mind to begin to undress. Occasionally in a small hospital we have heard, on inquiring for the night nurse's quarters: "I am sorry to say she has to sleep in a day nurse's room; it is an uncomfortable arrangement for both, but we cannot avoid it." We think the word "cannot" should not be uttered in connection with such an inexcusable custom. At one country hospital the night nurses' quarters are situated in the lodge at the entrance gates, which seems likely to be, on occasions, a somewhat lively spot for the purpose indicated.

Quiet, free ventilation and an even temperature are needful for night nurses' rooms, and there should always be good dark blinds provided to keep out the intruding daylight. This last point is a small one which is frequently entirely overlooked, and although nurses are often accounted as being somewhat addicted to grumbling, our own experience of them is that they are needlessly averse to calling attention to small personal discomforts. Night nurses should be well and consistently fed. Besides the regular meals, they should be encouraged to take a cup of hot milk or cocoa just as they go to bed, especially if they have had a walk, or are indifferent sleepers. Workers who declare they "hate night duty because there is so little to learn then," are hardly worth consideration, but to them we would recommend a little more humility. Wiser women, aye, and men too, say they are "always learning," and therefore we have scant pity to spare for the young lady who requires to be taught as if still in the school-room, whilst she shuts her eyes to the knowledge which awaits her on every side.

After all we should hardly look on sick people entirely as mere lesson books. In observing their needs we unconsciously learn how best to meet them, whilst in studying the symptoms of their diseases a nurse's only motive should be the relief of the patient, both by accurately following the doctor's orders and implicitly observing his lines of treatment.

Anyone who has suffered from serious illness, or who has nursed those dearest to her in mortal sickness, must always think of the long night hours with a certain amount of awe. Even in pleasant homes where comforts abound, the nights are only robbed of their terrors by human companionship. There is no loneliness in the day which can in any way touch that of the night for sick and suffering humanity.

It is terrible to think of institutions where, even at the present time, there is no trained nursing at night; where paupers pass through the last sad hours of their sad lives practically untended. In other places there are so few skilled nurses installed, each of whom has so large a charge that she can merely pay occasional short visits to poor creatures who really each of them need some hours of individual attention. Such things should not be possible. Even alas! in households where no pinch of poverty is felt, to "sit up all night" is often thought quite meritorious by the person who offers to do it. Frequently two members of the household waste their strength by sitting up together, and "keep each other company," but the patient generally fares better in the hands of a single attendant, whose attention she secures, and benefits greatly thereby.

It is not only in hospitals and other sick institutions that quiet is imperfectly secured for the weary night nurse, but in many a private wealthy household she finds the noisiest room in the mansion allotted to her use. We heard one say the other day, "Yes, the people were kind, but I got so little sleep, for the other children always played in the corridor, generally just outside my door. I mentioned it once or twice, but I was thought fanciful, so I just held my tongue and got snatches of sleep when those healthy little creatures happened to be elsewhere."

Perhaps the responsibilities of night work have also a deterrent effect on conscientious women; they feel acutely how much more they are "left to themselves" during the night than they are in the day. Although the doctor is under the same roof and a night superintendent supervises, emergencies must occur which demand immediate action. A good nurse is always prepared for surprises, and seldom loses her nerve; she does the best thing possible at the moment, but on night duty she misses the moral support which accompanies the same workduring the day.

If the young nurses who set their minds against night duty would pause awhile and consider that it is no worse for one than for another, and that much of their opposition is based upon purely selfish grounds, we think they would show a fairer spirit towards the work, and also towards their fellow-workers. Much might also be done in maintaining an average of good health if all nurses would feel it their plain duty to secure this possession for themselves by a wise indulgence in nature's gifts of fresh air, quiet sleep, and wise feeding.

### Everybody's Opinion.

[*Correspondence on all subjects is invited, but we cannot in any way be responsible for the opinions expressed by our correspondents. No communications can be entertained if the name and address of the correspondent is not given, or unless one side of the paper only be written on.*]

### A PORTABLE TANK.

"Policy 1,976" writes: I had a narrow tank made to hang over the frame-work of the doorway. Under this was a sheet with hem at top wide enough for the lath of a blind, which must be put flat, not edgeways as in a blind, a hank of wool cut through, wrung out of water, divided into four parts, then doubled, the doubled part put deep into the tank and the cut ends spread out on to the hem of sheet. This plan never fails, the irrigation going on successfully until the tank is empty, about every twelve to twenty-four hours according to the dryness of the atmosphere. The number of skeins of wool required being more or less according to the thickness of the wool. It is a very portable arrangement, and any private nurse can easily have it strapped to umbrella and rug as she goes from case to case.

### Notes and Queries.

### Queries.

(75) *Training.*—Where can I get trained as a male nurse?—*Edgar.*

(76) *Probationer.*—Will two years in a children's hospital, followed by one year in a general hospital, make me a thoroughly qualified nurse?—*E. E. W.*

(77) *Nurses for India.*—Particulars wanted by E. S.

(78) *Dr. James Anderson.*—Where can I learn some particulars of Dr. Anderson's life and work?—*Nurse Mary.*

(79) *Extension Shoe.*—Where can I get the shoe invented by Mr. E. O'Connor, described in The Hospital, February 11th.—*Auntie.*

(80) *Mental Nursing.*—Is there a book on this subject suited to nurses?—*Probationer.*

### Answers.

(75) *Training* (*Edgar*).—We do not know of any general hospital which trains male nurses. You had better apply to "Medical Superintendent, Berrywood Asylum, Northampton," for the terms on which pupils are taken there.

(76) *Probationer* (*E. E. W.*).—No. You must have three years in a general hospital if you wish for a certificate. Do not begin your training until you are 25, if you can avoid it. Probationers are admitted to some country hospitals and infirmaries younger than to metropolitan hospitals.

(77) *Nurses for India* (*E. S.*).—Kindly put your question more definitely, and we will try to answer it in this column.

(78) *Dr. James Anderson* (*Nurse Mary*).—The *Lancet* of March 11th gives a full and most interesting account of this gentleman.

(79) *Extension Shoe* (*Auntie*).—The address you require is that of E. O'Connor, Messrs. Lilley and Co., 275, High Holborn, London.

(80) *Mental Nursing* (*Probationer*).—"Lectures for Asylum Attendants," by Dr. Harding, published by the Scientific Press, 428, Strand.

### Our Examination Questions.

After receiving such a large number of answers, many of them excellent ones, to our Question for February, it is somewhat disappointing to find that neither in quantity nor quality do the replies to the March Question compare favourably with those of the previous month. We cannot select one sufficiently good for a prize or for publication. The subject is, and possibly may remain, an unfamiliar one to English nurses, but we wish more attempts had been made to send short and satisfactory answers. "Explain the duties and responsibilities of a nurse in charge of cholera patients" is not a hard problem to solve. We asked for the "nurse's duties" only, and we flattered ourselves that most of our readers would hold clear views as to these. We hope they do not scorn the question for its simplicity. As regards the answers which have come to us, most of them show that the writers fail to realise the urgency of the disease, the need of immediate treatment, and the generally short duration of an attack. Our correspondents give much attention to preliminary details, such as elaborate preparation of sick room, special toilets for the nurses; and also to the care of the patients during lengthy convalescence. Hardly anyone seems to understand that cholera is, as somebody wrote recently in The Hospital, an emergency, an d every qualified nurse ought therefore to understand how to deal with it, and should also be competent to teach other people what to do till the doctor comes. In fact, how to do the very best thing for the patient, for his friends, and for the community at large, as all three points are included in a nurse's "responsibilities," We were exceedingly pleased to find how thoroughly they were understood and acknowledged in the answers sent in February, and we are therefore encouraged to hope that it is not lack of knowledge which has made our nurse-readers rather bad correspondents this month. We trust they will send us plenty of good answers to the question for April, and we call their attention to the rules respecting these.

Examination Question for April.

"What are the special points to be observed in Making and Applying Poultices and Fomentations; and how often should either be changed when no special order is given?"

Answers must be sent in by April 4th. They must be clear and concise, written on one side of the paper only, accompanied by writer's name and address, and directed to "Nursing" Editor of The Hospital.

### Hints for Nurses.

A box of patterns from Egerton Burnett, of Wellington, Somerset, gives a definite direction to thoughts already straying towards seasonable clothing. Winter cloaks not only look shabby in the sunshine, but they become a weariness to the flesh, for everybody is conscious of feeling limp in the first eagerly-welcomed spring days. The packet of waterproof materials suitable for nurses' cloaks is therefore specially appropriate just now. The fine, light cloths of pure wool, in many shades of grey and blue as well as in black, are admirable for summer wear. There are also cloakings of medium thickness which can safely succeed our well-worn and valued winter wraps. In ordering materials nurses had better say whether they want them waterproofed or not, as Mr. Burnett supplies both kinds. Some specially fine blue serges for nurses' dresses are very tempting investments, for they are of double width and moderate price, pleasant to touch and to wear. An excellent variety of grey dress materials are also supplied by Mr. Burnett; they vary from the lightest to the darkest shades of that very useful colour, and are of different textures. Washing dresses, although we happen to refer to them last, must naturally take first rank in a nurse's eyes. "The Nightingale Stripe" is appropriate for everybody, from the toddling child to the trained nurse, because it is pretty and because it is strong. Another pattern shows a fine stripe of alternate pale blue and white, and is 29 in. wide and 10¾d. per yard, both sides alike, whilst a slightly wider grey and white stripe is only about half the price and is very useful wear. We are particularly struck with some dark blue and white stripes, which are certainly very professional-looking, and make most becoming and serviceable costumes. These materials are 27 in. in width, and are priced at 6½d. In these antiseptic days too much attention cannot possibly be paid to securing thoroughly good washing dresses, and we certainly all agree in preferring them to be as nice looking as possible.

### For Reading to the Sick.

QUICKENING.

No two things which are used for the same purpose can be more unlike, or give more different impressions to the beholder, than a quick-set hedge and a dead wall. Both are used to keep out trespassers and to keep property safe, but there the resemblance ends; for the very name, a quick hedge, suggests life everywhere, a fine breezy country and a bright cheerful scene, while the thing itself is delightful to the eye, whether it consists of thick hawthorn or stout glossy holly. Year after year it grows broader and higher, becoming fitter for defence, requiring only a little clipping here and a little trimming there, like all other earthly products, to make it perfect.

Alas! for a dead wall; who can express the melancholy monotony of having one always before you! To gaze constantly on rows of cemented bricks, to mark them getting dirtier and uglier the longer they remain, till at last they can stand no longer, and time and the builder clear them away, to be replaced possibly by something equally objectionable. Granted that there are walls of noble proportions, with handsome coped tops, which, when covered with grey and yellow moss, appeal to our sentimental admiration, even when bulging to their fall. Yet these are generally the boundary of some park-like domain and borrow their picturesqueness from their surroundings.

It is life and death, as their names denote, which make the difference between these two barriers, the quick hedge and the dead wall, and it is the same with our souls and bodies. To be quick, lively, full of energy—this is what we long for when sickness is upon us, and we can do nothing for ourselves. With what rapture do we hail the first flickering of returning health which gives us hope for the renewal of our life. We may be fixed to one spot, as the quick-hedge is, but we can grow and flourish and do our duty there as well as the mightiest tree which spreads its branches to the four winds of heaven.

Our souls, too, sink into despondency when they are dead to spiritual things. Let us at once pull down and clear away the blank wall of pride, or sullenness, or perversity, which we have built up between ourselves and our God and our fellow-creatures, and put in its place the beautiful quick-living partition, through which comes the cheering warmth of our sun, and the soft, invigorating breath of the Holy Spirit. Our hedge must have "Faith for its fixed, unswerving root" with Hope, blossoming like the delicate pink and white of the hawthorn or the scarlet berry of the holly for its unfading flower, and then the "deeds of Charity" will come naturally to be the glory of our bower. But as God alone can give the increase, let the planter and sower and cultivator be whom he may, we will ever put up the prayer of the Psalmist, "O quicken me in Thy righteousness," so that our leaf shall be always green, and we shall not fear when the heat of affliction cometh.

### London Hospital Training School for Nurses.

A course of lectures to the nursing staff of the London Hospital on Elementary Physiology and Medical Nursing, by Dr. Percy Kidd, M.D., F.R.C.P., will commence on Wednesday, March 22nd, at eight p.m. Tickets for the course can be obtained by nurses not belonging to the hospital on payment of a fee of 10s. 6d.

### The Muses' Looking Glass.

"The quotation of books, old and new, is the highest pleasure of a young author," says Heine, and proceeds to show how the thing may be done. The youth of an author cannot perhaps always be safely inferred from this prominent feature of a book. But here there are not wanting other indications. The freshness and enthusiasm, the directness of purpose, the energy with which mighty conversations are put through, and a certain simplicity of mind and motive, all bear the stamp of youth. And this is the charm of the book—a charm which bears the reader pleasantly forward and lands him at the end touched with admiration at the courage of the female students, and more strongly convinced than ever of the need for women doctors. A book which thus achieves its avowed purpose can be hailed as a success, and the author will, no doubt, in subsequent work, avoid flaws which would have contributed failure to less spirited writing.

Mona Maclean is a person of many fascinations and lovers. An orphan with £300 a year of her own, she studies medicine at University College, and the story opens with the news of her second failure in the intermediate examination. The reader perceives at once that she knows too much, and this fact becomes more and more crushingly apparent. Deeply absorbed in her scientific studies, she has, nevertheless, an easy acquaintance with books, botany, and bonnets. Her familiarity with German is always unpleasantly prominent; she quotes French when in sufficiently high society, and uses airy Spanish ejaculations; she sings in several styles; she plays lawn tennis and the piano, and sketches in water colours; she understands the art of flirtation, has a passion for dress, and a preference for dry champagne. Yet she is a pleasant person through it all, bearing up under this weight of talents better than might be supposed, chiefly by virtue of high spirits and a warm heart.

Disgusted with her second failure, she decides to accept an invitation from a cousin of her father's living in a remote Scotch watering-place, and retire for six months from the world of medicine. She then discovers that the cousin keeps a milliner's and "all sorts" shop in the village of Borrowness, but boldly determines not to draw back. Before beginning her exile, however, she passes a fortnight with her mother's relations, Sir Douglas and Lady Munro, in Norway, and from these high surroundings and a flirtation with an East Indian of distinction, she passes into her new life as shop assistant to the elderly cousin, whose vulgarity of mind and manners knows no disguise. The sketches of life in Borrowness are excellent, and the situations are worked out with considerable skill and humour. Bound by a promise not to reveal her medical studies, Mona falls into her position as cousin to the not very highly-respected Miss Simpson, and milliner in ordinary to the poorer farm girls and servants of the neighbourhood. She throws herself into shop-keeping and bonnet making with characteristic energy, and meets her fate in the person of a Dr. Dudley, preparing, like herself, for his intermediate examination at University College. The little romance flourishes in a kindly atmosphere of entire reserve on her side, and slowly vanishing doubts as to milliner's assistants on his. On both sides is a determination to postpone explanation till the examination is over. With these diversions, and with interludes of rejected addresses from the East Indian, and a more humble lover in the person of a botanical linen draper, the six months wear away, and Miss Maclean returns to Gower Street to pursue her medical studies with more regulated zeal. We get a funny little picture of the talk of the schools in this connection.

"The dissecting-room was abandoned by all save a few enthusiastic students who had not yet wholly exhausted the mysteries of Meckel's ganglion, the branches of the internal iliac or the plantar arch.

"'If you juniors want a piece of advice,' said the dissector of Meckel's Ganglion laying down her forceps, 'you may have it for nothing. Form a clear mental picture of the spheno-maxillary fossa. When you have that, the neck of anatomy is broken. Miss Warden, suppose first to refresh all our memories, you run over the foramina opening into the spheno-maxillary fossa and the structures passing through them.'"

But Miss Warden refuses, quoting in self-justification the ignorance of a certain Miss Clark. '"I was dissecting the popliteal space the other day and she asked me if it was Scarpa's triangle.' A general murmur of incredulity.

"She has not had an inferior extremity," said a young girl, "you can only learn your anatomy by dissecting yourself." It is a heavy price to pay," said she of the spheno maxillary fossa; and a difficult job at the best, I should fancy."

All this time Dr. Dudley has no conception of the real life of his abnormal milliner. They go through their examination in the same room in entire unconsciousness on his part, and as he is ignorant of her real christian name, he is not enlightened when the lists are out. Returning to Borrowness after all is over, he finds the inconvenient cousin off to America, and no trace of his charmer, only a dim rumour of her wealth and connections which sends him back to London with a sore impression of having been fooled. Not unnaturally he is considerably taken aback, too, when he sees her again the centre of admiring crowds bearing off the Physiology medal at Burlington House. The experienced reader perceives that a bridge will be found sooner or later over the awkward little rift which divides the two learned doctors. We may be permitted to quote the conclusion.

"They had returned from the Continent to Gower-street, a few hours before. Dr. Dudley had scarcely finished reading his first letter when a patient was announced, and a moment later a young girl entered the room with a shrinking uncertain step. Her hair was wet with the rain, and her white face expressionless, save for its misery.

"'Do you wish to consult me?' he said, 'Sit down, what can I do for you?'

"She looked at him for a moment, and tried to speak, but her full lips quivered, and she burst into hysterical tears. His practised eye ran over her figure half unconsciously. 'I think,' he said kindly, 'you would rather see the doctor who shares my practice,' and he rose and opened the door. Mona looked up smiling. She was sitting alone in the firelight, and his heart glowed within him as he contrasted her bright, strong, womanly face with that other. 'Mona, dear,' he said quietly,' 'here is a case for you.'"

* "Mona Maclean, Medical Student." By Graham Travers. 3 vols. (Blackwood and Sons.)

### Death in our Ranks.

Nurse Amson, for some years parish nurse for the Lytham and St. Anne's Parish Nurse Committee, died on February 28th after a lingering illness. Nurse Amson was greatly beloved by her numerous patients.

Nurse Hanney Jean Hughes died at Fulham Infirmary on February 25th after a few days' illness, greatly regretted.

Miss Mabel Wynn, aged 18, a probationer at the Victoria Hospital for Children, Hull, has died of pneumonia following an attack of measles contracted in the discharge of her duties. Miss Wynn gave every promise of attaining distinction in her profession, and her untimely death is greatly deplored.

Florence Lillian Hurt, of Wandsworth Infirmary, died on March 10th, aged 20, of typhoid fever and pneumonia, at the Fever Hospital, Stockwell.

### Wants and Workers.

A lady will be glad to know how she can get a child admitted into a Home for Imbeciles. Its parents can only pay a very small sum towards its support.—*Vera.*

### The Book World for Women and Nurses.

[We invite Correspondence, Criticism, Enquiries, and Notes on Books likely to interest Women and Nurses. Address, Editor, THE HOSPITAL (Nurses' Book World), 428, Strand, W.C.]

### REVIEWS FOR MARCH.

TERRIBLE revelations appear in Lady Violet Greville's article in the NATIONAL REVIEW on the "Victims of Vanity." "What can we think of the heroism of the girl who determined to reduce her waist to a maximum of sixteen inches and a-half, cheerfully deprived herself not only of dainties, but even of necessary food, lived the severe regimen of a jockey, took exercise early and late, and finally slept in her corsets? She reduced herself from half to three-quarters of an inch every month. An 'artiste en corsets' solemnly avers that she has the tightest lacing clientele in London; that she makes stays of all shapes and sizes, from diaphanous satin to rude coutil, for all times and seasons, for rowing, riding, dancing, and even bathing! All is vanity, even the waist of the bather. Further, she deposed that the smallest corset she had ever made was twelve inches and a-half. The assistant young lady even appeared for a few moments with a waist of thirteen inches and a-quarter. Smilingly, the maiden explained that she could not bear this compression for more than half an hour." The whole article gives food for much sorrowful reflection, even if it is calculated to rouse in our busy and healthy readers something of the self-gratulatory spirit of the Pharisee.

MISS CLEMENTINA BLACK, in the NINETEENTH CENTURY, advocates the organization of a brigade of really good out-door servants. She traces the dislike to domestic service to the system of "total personal subservience" prevailing, and sees the remedy in a state of things where servants will cease to be domiciled under their employer's roof, and must instead come to work for a specified number of hours. The necessary increase of expense to the employer, the danger of infection from insanitary homes, and the horrors of over-crowding to which young women are exposed in their own families, are points which Miss Black does not touch upon. It is a pleasant assumption that young servants all come from homes of good and wholesome influence, but it is, unhappily, not based upon fact.

IN the CONTEMPORARY REVIEW we have an article of the familiar Women's Rights order from Miss Mary Aldis, deprecating the exclusion of women from certain forms of labour in Australia and New Zealand and the reservation of University endowments to men. That the great majority of men have wives and families dependent on their earnings, and are, to some extent, justified in protesting against the lowering of wages by an influx of cheap female labour, is a point which is often lost sight of in dealing with this vexed question. Many women who rush thoughtlessly at men's occupations, aim only at earning a little money for personal expenses, and these women aid indirectly in forcing to emigration the men whose proper vocation should be to support them in the comfort of a home life.

THE WESTMINSTER REVIEW devotes much space this month to pleas for the helpless. A very forcible exposure of the twin evils of baby farming and infant insurance is headed "Moloch in England." The facts collected by C. R., and the statistics of the Society for the Prevention of Cruelty to Children, are terribly significant. In the cases of cruelty to children with which it dealt during twelve months, 1,298 of the

### THE BOOK WORLD FOR WOMEN AND NURSES—*continued.*

children were found to be insured at the average rate of £4 12s. 8d. a-head. In the cases of the following year 3,809 were found to be insured. "Where the system of insuring children is most common the mortality is highest. We should, therefore, unhesitatingly say let child insurance be abolished by law." An equally mournful array of facts is marshalled by M. S. C. Crawford in her paper on "The Maltreatment of Wives," facts which are, alas! too well known to need more than a passing reference. In "A Plea for Women," a woman urges on behalf of some of her fallen sisters that they are forced into the pitfalls which lie in their path by ignorance, desperation, or lastly, by a want of self-control, the necessity for which is too often overlooked by the educators of youth.

Mrs. Garrett Anderson gives a concise history in the Fortnightly Review of the movement in support of the admission of women into the medical profession. It seems that Miss Elizabeth Blackwell, of New York may claim the honour of being the pioneer in this movement, having, in the face of many difficulties, succeeded in 1849 in obtaining the degree of M.D. from the University of Geneva, U.S.A. After tracing the continuous progress of the last forty years, Mrs. Anderson points out that medical women have hitherto been excluded from all the medical societies in London, and that it is difficult to exaggerate the disadvantage which results from such exclusion. She concludes with the hope that at no distant date it will be recognised that women doctors have done good service to the community at large as well as to themselves.

### LITERARY INTELLIGENCE.

We understand that Miss Hampton, the Lady Superintendent of the Johns Hopkins Hospital, Baltimore, has just completed an exhaustive work on Nursing. Miss Hampton is so able a woman, and has proved by her administration at the Johns Hopkins Hospital that she possesses so rare a knowledge of every section of the nursing field, that we are confident her book will excite much interest and attract an army of readers. It will be published, we understand, at the end of April or beginning of May next.

Mothers who take a genuine interest in their children should procure a little book entitled Schoolboy Morality; an Address to Mothers (Elliot Stock, 62, Paternoster Row, E.C.) This booklet deals with a difficult and delicate subject in a way which must commend itself to the acceptance of all mothers throughout the country. There can be no doubt that the rising generation would be greatly benefited were they to be taught at an early age the elements of physiology and made to realise, in more ways than one, how they are made. Schoolboy morality would be largely promoted by such instruction, and this address to mothers, now in its twenty-eighth thousand, will help materially to aid reforms which are sadly needed in this country at the present day.

We have received some early copies of a new journal of civic and social progress, entitled London. Its aim seems to be to deal with all questions included in the municipal administration of the metropolis of the Empire. So far London seems calculated to fulfil many useful purposes, but we hope it may be possible for its conductors to give fuller reports of the meetings, not only of the London County Council, but of its committees, and also of similar bodies charged with local government within the metropolitan area.

